# A Comparison of Tumor Tracking Accuracy Using Real-Time Adaptive Motion Management on Helical Versus Robotic Radiotherapy Platforms: An Interdisciplinary Study

**DOI:** 10.7759/cureus.84956

**Published:** 2025-05-28

**Authors:** Shishir Kumar, Dhiren K Panda, Parveen Sharma

**Affiliations:** 1 Anatomy, Institute of Medical Sciences (IMS) and SUM Hospital, Siksha 'O' Anusandhan University, Bhubaneswar, IND; 2 Dentistry, Tantia University, Sri Ganganagar, IND

**Keywords:** cyberknife, helical radiotherapy, precision oncology, radiotherapy, real-time adaptive motion management, robotic radiotherapy, tomotherapy, tumor motion, tumor tracking

## Abstract

Background

Real-time adaptive motion management (RTAMM) systems have enhanced the accuracy of tumor tracking during radiotherapy. This study aimed to investigate the tumor tracking accuracy of helical tomotherapy and robotic CyberKnife systems. The study objective was to assess the tumor tracking accuracy of RTAMM in helical and robotic radiotherapy systems and determine their clinical relevance in treating tumors with complicated motion.

Methodology

This retrospective study analyzed the clinical and imaging data of 60 patients with lung or liver tumors treated with TomoTherapy or CyberKnife between March 2024 and January 2025. Tumor positional deviations were tracked and recorded in real time using onboard imaging. Mean deviations and standard deviations were calculated, and independent t-tests were used to compare tracking accuracy.

Results

CyberKnife demonstrated superior tumor tracking accuracy, with a mean deviation of 0.8 mm (±0.2 mm) versus 2.3 mm (±0.4 mm). CyberKnife outperformed tomotherapy in tumors exhibiting high motion, with a statistically significant difference (p < 0.01).

Conclusions

Robotic CyberKnife systems provide more accurate tumor tracking than helical tomotherapy systems, particularly for tumors with complex motions, validating their use in precision radiation therapy.

## Introduction

Accurate tumor localization and motion management are essential components of modern radiotherapy, particularly for tumors located in regions subject to significant physiological motion, such as the lung and liver. Respiratory-induced tumor motion poses a considerable challenge in achieving precise dose delivery, as even small deviations can lead to underdosing of the target or overdosing of adjacent healthy tissues [1-3]. Consequently, technological advances in image-guided radiotherapy (IGRT) and adaptive motion tracking have become central to optimizing therapeutic outcomes while minimizing toxicity.

Among the current radiotherapy technologies, helical TomoTherapy and robotic CyberKnife systems represent two distinct approaches to managing intrafractional tumor motion. TomoTherapy delivers continuous helical intensity-modulated radiation therapy (IMRT) and generally relies on pretreatment imaging, such as four-dimensional computed tomography (4D-CT), to model the respiratory motion. While effective in some scenarios, this predictive strategy does not allow for real-time motion correction during dose delivery [4,5].

In contrast, the CyberKnife system integrates real-time imaging with robotic delivery. It uses fiducial markers and continuous X-ray imaging to dynamically track and adjust the beam to the tumor’s position throughout the treatment session. This enables submillimeter tracking precision and compensates for translational, rotational, and deformation-related movements [6-8]. These real-time adaptive capabilities are especially critical in stereotactic body radiation therapy (SBRT), where high-dose hypofractionated regimens demand high spatial accuracy.

Although both systems have demonstrated clinical efficacy, there are limited comparative data on their performance, specifically in the context of tumor tracking accuracy under real-time adaptive motion conditions. Understanding the relative strengths and limitations of these platforms is essential for guiding clinical decision-making and system selection, especially in centers with access to both.

This study aimed to compare the tumor tracking accuracy of helical TomoTherapy and robotic CyberKnife systems in patients with thoracoabdominal tumors. By analyzing intratreatment positional deviations, we sought to determine which platform offers superior motion compensation and to evaluate the clinical implications of their respective tracking capabilities.

## Materials and methods

Study design

This retrospective comparative study included 60 patients with thoracoabdominal tumors treated between March 2024 and January 2025. The study cohort included patients diagnosed with lung or liver malignancies who underwent radiotherapy using either the helical TomoTherapy or CyberKnife systems. Patients were assigned to the following two groups based on the treatment system: 30 patients in the TomoTherapy group and 30 in the CyberKnife group.

Patient selection

The inclusion criteria comprised adult patients (≥18 years) with pathologically confirmed solid tumors located in the lung or liver, planned for curative-intent radiotherapy, and with available imaging data suitable for motion tracking analysis. Patients with prior irradiation to the same anatomical site, missing tracking data, or contraindications to fiducial placement (for CyberKnife) were excluded from the study.

Treatment delivery and motion management

TomoTherapy Group

Patients in this group underwent simulation using 4D-CT to capture tumor motion over the respiratory cycle. Motion management was based on internal target volume (ITV) expansion derived from 4D-CT data. No intrafraction tracking system was employed during dose delivery. IGRT is limited to pretreatment megavoltage computed tomography (MVCT) imaging for patient alignment. Treatments were delivered using helical IMRT with fixed beam modulation without Synchrony or SGRT integration [3].

CyberKnife Group

Patients treated with CyberKnife underwent fiducial marker implantation under ultrasound or CT guidance before simulation. Tumor motion was tracked in real-time using the Synchrony respiratory tracking system, which integrates continuous X-ray imaging and robotic arm compensation. The tumor position was monitored in six degrees of freedom, and the beam delivery was dynamically adjusted throughout the treatment to maintain submillimeter accuracy [4,5].

Tumor tracking data collection

Positional tracking data for each patient were retrieved from the system log files and treatment records. In the TomoTherapy group, deviations were calculated by comparing the planning ITV positions with the daily MVCT-derived tumor positions. In the CyberKnife group, deviations were automatically recorded during real-time tracking and included time-stamped positional coordinates along all translational axes. For each patient, the mean and standard deviation of the intrafractional tumor displacement were computed.

Evaluation of tracking accuracy

Tracking accuracy was defined as the absolute deviation (in mm) between the planned and observed tumor positions during treatment. The primary outcome was the average intrafractional tumor deviation per patient per system. Tracking errors along the superior-inferior (SI), anterior-posterior (AP), and lateral (LAT) axes were analyzed.

Statistical analysis

Descriptive statistics (mean and standard deviation) were calculated for positional deviations within each group. The normality of the data was assessed using the Shapiro-Wilk test. Group differences were analyzed using independent two-tailed t-tests for normally distributed variables and the Mann-Whitney U test for non-parametric data. Statistical significance was set at p-values <0.05. Statistical analyses were conducted using SPSS version 27 (IBM Corp., Armonk, NY, USA).

Ethical approval

This study was approved by the Tantia University, Sri Ganganagar Institutional Ethical Committee (approval number: TU/IEC/2025/16). Patient data were anonymized, and no direct patient contact occurred. This study adhered to the principles of the Declaration of Helsinki.

## Results

This study included 60 patients who were evenly split between lung (n = 30) and liver (n = 30) tumors. Tumor positional deviations were tracked in real time during treatment using onboard imaging on both radiotherapy platforms.

Tumor tracking accuracy

The CyberKnife system demonstrated superior tumor tracking accuracy, with a mean positional deviation of 0.8 mm (±0.2 mm), compared to 2.3 mm (±0.4 mm) for the TomoTherapy system. This difference was statistically significant (p < 0.01) (Table 1).

**Table 1:** Tumor tracking accuracy comparison between the helical TomoTherapy and robotic CyberKnife systems. The robotic platform showed significantly superior accuracy.

System	Mean accuracy (mm)	Standard deviation (mm)	Number of patients
Helical radiotherapy (TomoTherapy)	2.3	0.4	30
Robotic radiotherapy (CyberKnife)	0.8	0.2	30

Positional deviation in lung and liver tumors

To further explore the platform’s capabilities, Table 2 presents the positional deviation for lung and liver tumors separately. In patients with lung tumors, the CyberKnife system maintained a mean deviation of 0.6 mm (±0.1 mm), compared to the TomoTherapy system’s mean of 2.1 mm (±0.5 mm). In liver tumors, the CyberKnife system achieved a mean deviation of 1.0 mm (±0.2 mm), while the TomoTherapy system showed a higher deviation of 2.4 mm (±0.3 mm). The differences in tracking accuracy between the systems were consistent across both tumor types, further demonstrating the superior performance of the robotic system.

**Table 2:** Tumor tracking accuracy stratified by tumor type. The robotic system demonstrated higher precision in both lung and liver tumors, particularly in those with complex motion patterns.

Tumor type	System	Mean accuracy (mm)	Standard deviation (mm)
Lung tumors	Helical radiotherapy (TomoTherapy)	2.1	0.5
	Robotic radiotherapy (CyberKnife)	0.6	0.1
Liver tumors	Helical radiotherapy (TomoTherapy)	2.4	0.3
	Robotic radiotherapy (CyberKnife)	1.0	0.2

Impact of tumor motion on tracking accuracy

Tumor motion plays a pivotal role in the accuracy of radiation delivery. Table 3 presents the deviation results based on tumor motion severity, categorized into low, medium, and high motion tumors. In low-motion tumors, both systems showed similar performance. However, in medium and high-motion categories, the CyberKnife system demonstrated far superior tracking, with average deviations of 0.5 mm (±0.2 mm) and 0.9 mm (±0.3 mm), respectively, compared to the TomoTherapy system, which recorded deviations of 2.0 mm (±0.6 mm) and 2.8 mm (±0.5 mm), respectively.

**Table 3:** Tumor tracking accuracy based on tumor motion severity. The robotic CyberKnife system showed a distinct advantage in tracking tumors with high motion.

Tumor motion	System	Mean accuracy (mm)	Standard deviation (mm)
Low motion	Helical radiotherapy (TomoTherapy)	1.9	0.4
	Robotic radiotherapy (CyberKnife)	0.7	0.2
Medium motion	Helical radiotherapy (TomoTherapy)	2.0	0.6
	Robotic radiotherapy (CyberKnife)	0.5	0.2
High motion	Helical radiotherapy (TomoTherapy)	2.8	0.5
	Robotic radiotherapy (CyberKnife)	0.9	0.3

Statistical comparison of deviations between systems

To further substantiate the significant difference in tracking accuracy, Table 4 presents a statistical breakdown of the positional deviations between the two systems. The CyberKnife system consistently demonstrated superior tracking accuracy compared with the TomoTherapy system, particularly in high-motion anatomical regions, such as the lung and liver. Independent t-tests revealed statistically significant differences (p < 0.01), confirming the high precision of the robotic system.

**Table 4:** Statistical comparison of tumor tracking accuracy. Independent t-tests were performed between the two radiotherapy systems. A significant difference was found (t = 19.78, p < 0.01), indicating superior tracking precision with the CyberKnife system.

System	Mean positional deviation (mm)	95% confidence interval (mm)	t-score	P-value
TomoTherapy (helical)	2.3	2.0–2.6	19.78	<0.01
CyberKnife (robotic)	0.8	0.6–1.0		

## Discussion

Comparison of tumor tracking accuracy

This study provides compelling evidence that the robotic CyberKnife radiotherapy system delivers significantly superior tumor tracking accuracy compared with the helical TomoTherapy platform during real-time adaptive motion management (RTAMM). Our findings demonstrate a mean tumor positional deviation of 0.8 mm (±0.2 mm) for CyberKnife versus 2.3 mm (±0.4 mm) for TomoTherapy, consistent with prior studies highlighting CyberKnife’s precision due to its continuous intrafraction imaging and robotic beam adjustment capabilities [1,5,6].

The superior performance of the CyberKnife is primarily due to its advanced real-time tracking technology, which utilizes implanted fiducial markers and continuous X-ray imaging. This system dynamically compensates for complex tumor motions, including translations and rotations, by adjusting the radiation beam with robotic arms during treatment delivery. This flexibility is especially advantageous for tumors in thoracoabdominal regions, where respiratory-induced motion and organ deformation present substantial challenges [7,8].

In contrast, the TomoTherapy system relies mainly on pretreatment 4D-CT imaging and predictive motion modeling without routine intrafraction motion verification, unless supplemented by additional technologies such as Synchrony or SGRT, which were not used in this study. While TomoTherapy provides excellent dose conformality and is effective for tumors with predictable motion patterns, it lacks the capacity for real-time motion correction during radiation delivery, which explains the higher tracking errors observed in this study. This limitation aligns with existing literature emphasizing the challenges helical IMRT faces in rapidly compensating for irregular tumor movement [9,10].

Clinical implications

Clinically, the significantly enhanced tracking precision of the CyberKnife has important implications. Maintaining submillimeter accuracy in tumors with complex motion can improve the therapeutic ratio by maximizing tumor dose delivery while sparing the surrounding healthy tissues. This is particularly critical for lung and liver tumors, where motion-induced uncertainties can compromise the treatment efficacy and increase toxicity risks. Thus, selecting a radiotherapy platform capable of adaptive real-time tracking tailored to tumor motion characteristics can lead to improved patient outcomes and safety [11-13].

Patient compliance and treatment tolerability

Patient compliance and comfort further influence the accuracy of treatment. The frameless and non-invasive design of the CyberKnife reduces patient discomfort and minimizes involuntary movements, thereby enhancing tracking precision. Conversely, TomoTherapy’s longer treatment times and rigid immobilization may lead to increased patient fatigue and micro-movements, which can degrade the positional accuracy. These factors highlight the importance of considering patient experience alongside technological capability when optimizing motion management [14,15].

Practical challenges and limitations

Despite these advantages, implementing CyberKnife presents practical challenges, including high costs, operational complexity, and the need for specialized training, which may limit its accessibility in resource-constrained settings. Additionally, the requirement for continuous real-time imaging raises concerns regarding cumulative radiation exposure, necessitating rigorous quality assurance protocols to ensure patient safety [15,16].

Our study also benefits from interdisciplinary collaboration, incorporating anatomical verification and deformable image registration, which enhances the precision of motion tracking by accounting for organ deformation and rotational movement. This integrative approach underscores the value of combining radiological and anatomical expertise in advancing adaptive radiotherapy techniques [11].

Study limitations and future directions

The retrospective nature of this study and its relatively small sample size are limitations that may affect the generalizability of our findings to the general population. Moreover, long-term clinical outcomes were not assessed. Therefore, while the results provide important preliminary evidence favoring robotic systems for managing complex tumor motion, prospective studies with larger cohorts and extended follow-ups are needed to validate and expand upon these conclusions.

## Conclusions

The CyberKnife robotic radiotherapy system outperformed the helical TomoTherapy system in terms of real-time tumor tracking accuracy, particularly for tumors exhibiting intricate motion patterns. These findings advocate for the personalized selection of motion management strategies based on tumor- and patient-specific factors, potentially improving the precision and safety of modern radiotherapy.
